# Comparison of the Copan eSwab System with an Agar Swab Transport System for Maintenance of Fastidious Anaerobic Bacterium Viability

**DOI:** 10.1128/JCM.03246-15

**Published:** 2016-04-25

**Authors:** Kerin L. Tyrrell, Diane M. Citron, Eliza S. Leoncio, Ellie J. C. Goldstein

**Affiliations:** a R. M. Alden Research Laboratory, Culver City, California, USA; b UCLA School of Medicine, Los Angeles, California, USA

## Abstract

We compared the eSwab system to a swab with an anaerobic transport semisolid agar system for their capacities to maintain the viability of 20 species of fastidious anaerobes inoculated on the bench and held at ambient or refrigerator temperature for 24 or 48 h. On average, both systems maintained similar viabilities among analogous groups of organisms at both temperatures, although there were quantitative differences among some species.

## TEXT

Suitable specimen transport from collection to the laboratory is essential for accurate laboratory diagnostics. Given increasing laboratory centralization, transport times have increased as well, requiring systems to be robust enough to ensure sufficient organism collection, viability, and release. Specimens with anaerobic organisms have the added requirement of anaerobiosis for at least 48 h. The eSwab (Copan Diagnostics, Inc., Murrieta, CA) is a relatively new system compared to conventional gel-tube systems, and it lends itself to automation. The eSwab consists of a nylon-flocked swab, which provides better capillary action and stronger hydraulic uptake of liquids than do spun-fiber nylon or rayon swabs (1), and a screw-top tube containing liquid modified Amies medium. After specimen collection, the swab is inserted into the tube, and the scored shaft of the swab is easily broken to the length of the tube. A swab capture system in the cap locks the broken shaft into the lid of the tube after it is fully closed. Release studies that compared the flocked swab to conventional rayon or Dacron swabs have been performed (2), with favorable results, as have other studies that compared the viability of aerobic organisms and a small number of anaerobic organisms (1, 3–7). The recommended CLSI standard control strains have been shown in a previous study (1) to meet the requirements of the M40-A recommendations for transport systems (8). To our knowledge, this is the first study to compare numerous fastidious anaerobic bacteria. We compared the eSwab to Anaerobic Transport Medium (ATM; Anaerobe Systems, Morgan Hill, CA), both of which use a modified Amies medium in liquid or gel form, respectively, for the release and recovery of fastidious anaerobic bacteria from the swabs after 24 or 48 h at 4°C and room temperature (RT).

### Materials and methods.

Twenty fastidious anaerobes, nine Gram-positive and 11 Gram-negative organisms, from various sources were selected for the study (Table 1). The organisms were identified by standard (9, 10) or molecular methods. This feasibility study of the recovery of various fastidious anaerobic bacteria was based on the CLSI document M40-A (8), which is the approved standard for quality control of transport media. A 24- to 48-h subculture of each organism was suspended in saline in the anaerobe chamber to a turbidity of a 0.5 McFarland standard (∼1.5 × 10^8^ CFU/ml). To mimic clinical settings, the inoculation suspension was transferred to room air, and 0.1-ml aliquots were pipetted into microcentrifuge tubes to inoculate eSwabs and rayon swabs for the ATM system. Each system was set up for recovery testing at room temperature and 4°C; each temperature had separate tubes set up for subculture at *t* = 0, 24, and 48 h. At each sampling time, a suspension was made from each tube. The eSwab tube was vortexed for 5 s; the rayon swabs were removed from the ATM, and the tip was placed in 0.9 ml of saline and vortexed for 5 s. Each suspension was serially diluted, plated onto Brucella agar, and incubated in an anaerobic chamber for 24 to 72 h at 37°C, and colony counts were determined. The inoculum suspension was also serially diluted, and colony counts were performed. Although the CLSI M40-A quality-control standard recommends dilutions in triplicate and platings in duplicate, because this was a performance study of each transport system and not a quantitative quality-control analysis, each organism was studied once and each dilution was plated once. However, if the colony counts from the serial dilutions were inconsistent, the procedure was repeated. In addition, Clostridium difficile dilutions were also plated onto cycloserine-cefoxitin fructose agar with horse blood and taurocholate (HT) to better recover spores, which germinate more effectively in the presence of taurocholate (11).

**TABLE 1:** Specimen sources of fastidious anaerobic bacteria tested

Organism	Source
Gram negative	
Bacteroides fragilis	Appendix
Bacteroides thetaiotaomicron	Gluteal abscess
Bilophila wadsworthia	Appendix
Fusobacterium necrophorum	Tonsillar abscess
Fusobacterium nucleatum (1)	Facial lesion
Fusobacterium nucleatum (2)	Appendix
Porphyromonas asaccharolytica	Diabetic foot
Porphyromonas gingivalis	Tongue
Prevotella buccae	Abdominal abscess
Prevotella intermedia	Respiratory, sinus
Prevotella melaninogenica	Sputum
Gram positive	
Finegoldia magna	Respiratory, sinus
Parvimonas micra	Respiratory, sinus
Peptostreptococcus anaerobius	Unknown
Eggerthella lenta	Peri-rectal abscess
Propionibacterium acnes	Facial acne
Clostridium spp.	
Clostridium clostridioforme	Gluteal abscess
Clostridium difficile (1), nontoxigenic	Stool
Clostridium difficile (2), ribotype BI	Stool
Clostridium ramosum	Blood

### Release of sample from swabs.

**FIG 1:**
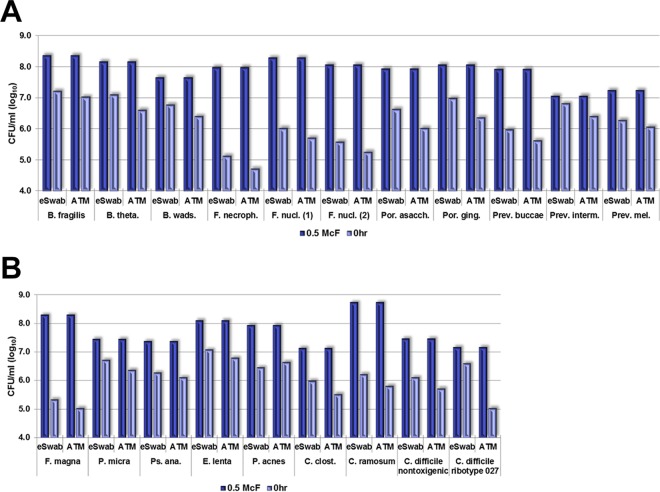
Release of inoculum by the eSwab and ATM systems. B. fragilis, Bacteroides fragilis; B. theta., Bacteroides thetaiotaomicron; B. wads., Bilophila wadsworthia; F. necroph., Fusobacterium necrophorum; F. nucl., Fusobacterium nucleatum; Por. asacch., Porphyromonas asaccharolytica; Por. ging., Porphyromonas gingivalis; Prev. buccae, Prevotella buccae; Prev. interm., Prevotella intermedia; Prev. mel., Prevotella melaninogenica; McF, McFarland standard.

The eSwabs released more organisms than did the rayon swabs, although, on average, the difference was minor (Table 2). There were some exceptions (Fig. 1). In the Gram-negative group, the eSwabs and rayon swabs retained 1.5 and 1.9 log_10_ CFU/ml on average, respectively. All Fusobacterium spp. were retained ∼1 log_10_ CFU/ml more than the Gram-negative group average by both swab systems. In the Gram-positive group, the eSwabs and rayon swabs retained 1.5 and 1.6 log_10_ CFU/ml on average, respectively. Finegoldia magna was retained by both swab systems ∼1.5 log_10_ CFU/ml more than the Gram-positive group average. In the Clostridium spp. group, the eSwabs and rayon swabs retained 1.4 and 2.1 log_10_ CFU/ml on average, respectively. Clostridium ramosum was retained by 0.7 log_10_ CFU/ml more with the eSwab and 1.6 log_10_ CFU/ml more with the rayon swab compared to the Clostridium spp. group average.

**TABLE 2:** Aggregate change

Organism and time (h)	Aggregate change in:					
	eSwab [CFU/ml (log_10_)]			ATM [CFU/ml (log_10_)]		
	Inoculum^*a*^	RT^*b*^	4°C	Inoculum	RT	4°C
Gram negative (*n* = 11)						
0	−1.5			−1.9		
0–24		−0.4	−0.6		−0.9	−0.6
24–48		−0.7	−0.2		−0.4	−0.3
Gram positive (*n* = 5)						
0	−1.5			−1.6		
0–24		0.1	0.1		−0.8	−0.5
24–48		−0.1	−0.3		−0.2	−0.3
Clostridium spp. (*n* = 4)						
0	−1.4			−2.1		
0–24		−1.2	−1.2		−1.0	−1.2
24–48		−0.3	−0.4		0.2	−0.1

a Inoculum loss by organism retention of swab.

b RT, ambient temperature.

### Recovery of sample.

**FIG 2:**
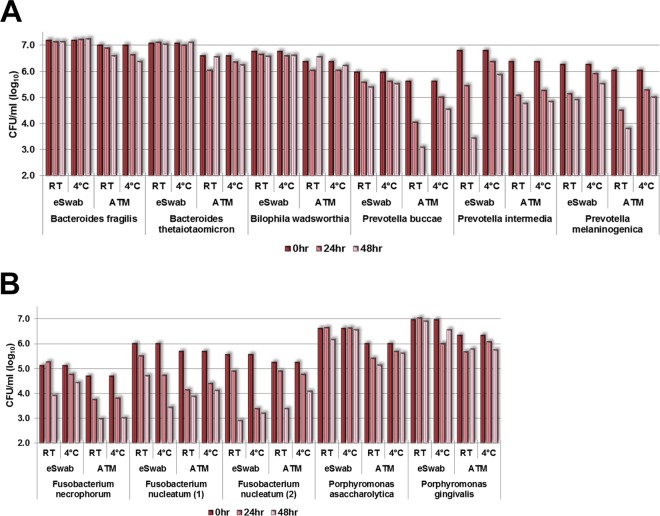
Recovery of sample at *t* = 0, 24, and 48 h (log_10_ CFU/ml), Gram-negative group. P. micra, Parvimonas micra; Ps. ana., Peptostreptococcus anaerobius; E. lenta, Eggerthella lenta; P. acnes, Propionibacterium acnes; C. clost., Clostridium clostridioforme; C. ramosum, Clostridium ramosum.

**FIG 3:**
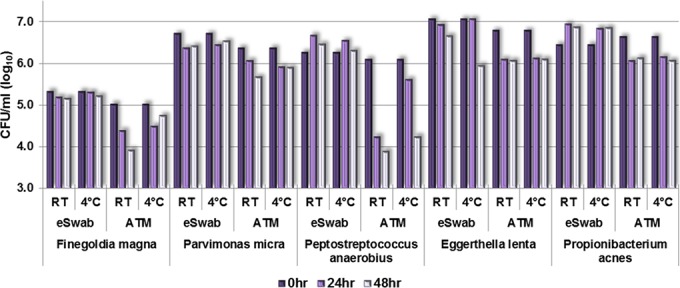
Recovery of sample at *t* = 0, 24, and 48 h (log_10_ CFU/ml), Gram-positive group.

**FIG 4:**
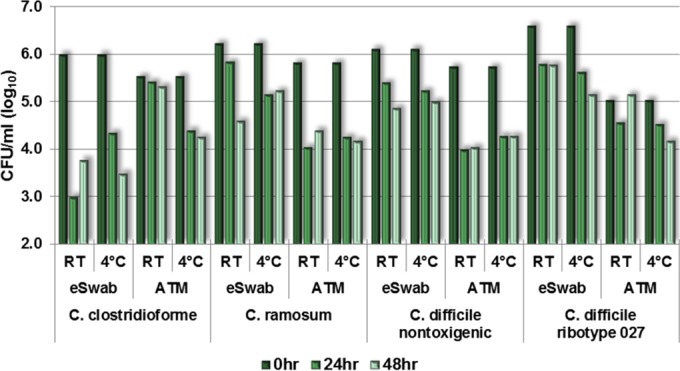
Recovery of sample at *t* = 0, 24, and 48 h (log_10_ CFU/ml), Clostridium group.

All organisms were recovered at room temperature (RT) and at 4°C at *t* = 0, 24, and 48 h (Fig. 2–4). Overall, both Gram-positive and Gram-negative organisms maintained similar average viabilities in both systems at RT and 4°C (Table 2); however, there were some exceptions.

In the Gram-negative group (Fig. 2), the best recoveries of all organisms over *t*_0–24 h_ and *t*_24–48 h_ at 4°C and RT were Bacteroides spp. and Bilophila wadsworthia, with an average loss of only 0.1 log_10_ CFU/ml over 48 h.

At 24 h, Fusobacterium necrophorum lost 0.9 log_10_ CFU/ml in ATM at 4°C and RT but had almost no loss in the eSwab. At 48 h, there was a 0.8 log_10_ CFU/ml loss in ATM at 4°C and RT, but, in the eSwab, there was a loss of 1.4 log_10_ CFU/ml at RT but only 0.3 log_10_ CFU/ml at 4°C. Best performance for F. necrophorum was the eSwab at 4°C. There were mixed results for the two Fusobacterium nucleatum species. One strain lost >1 log_10_ CFU/ml at 24 h in both systems and at both temperatures; the loss was less at 48 h for the eSwab at RT and for the ATM at 4°C and RT, but the eSwab lost >1 log_10_ CFU/ml at 4°C. The other F. nucleatum strain lost an average of 0.5 log_10_ CFU/ml in the eSwab at RT and ATM at 4°C and RT but lost 2.2 log_10_ CFU/ml in the eSwab at 4°C. Fusobacteria had the most loss in the Gram-negative group in both systems.

Porphyromonas asaccharolytica and Porphyromonas gingivalis had <1 log_10_ CFU/ml loss in both systems and at both temperatures over 48 h despite their fastidious nature.

On average, the Prevotella species lost the most during the first 24 h, 0.9 log_10_ CFU/ml for *t*_0–24 h_ and 0.5 log_10_ CFU/ml for *t*_24–48 h_. After 48 h, Prevotella buccae decreased only 0.5 log_10_ on average at 4°C and RT in the eSwab but, in the ATM, lost 2.5 log_10_ CFU/ml at RT and 1.1 log_10_ CFU/ml at 4°C. Prevotella melaninogenica lost 2.2 and 1.0 log_10_ CFU/ml in the ATM at RT and 4°C; the eSwab loss was 1.3 and 0.7 log_10_ CFU/ml at RT and 4°C. Prevotella intermedia performed similarly to P. melaninogenica, except that the eSwab loss at RT was 3.3 log_10_ CFU/ml. The best performance for all Prevotella species was the eSwab at 4°C.

In the Gram-positive group (Fig. 3), the average loss was 0.3 and 0.2 log_10_ CFU/ml at *t*_0–24 h_ and *t*_24–48 h_, respectively. The two systems performed similarly at RT and 4°C, with the exception of Peptostreptococcus anaerobius, which lost 2.2 and 1.9 log_10_ CFU/ml at RT and 4°C, respectively, over 48 h.

Clostridium spp. varied considerably (Fig. 4). Those strains known for producing more spores (e.g., C. difficile, ribotype 027) lost less in the first 24 h than in the second 24 h. The average loss of sample of Clostridium clostridioforme and C. ramosum was 1.1 log_10_ CFU/ml at *t*_0–24 h_, and there was no average loss at *t*_24–48 h_. The average loss of sample of C. difficile ribotype 027 was greater at *t*_24–48 h_ than at *t*_0–24 h_. C. clostridioforme did not perform as well in the eSwab system at *t*_0–24 h_ and *t*_24–48 h_.

All counts were higher on HT than on Brucella agar, with the exception of the 027 ribotype of C. difficile, indicating more organism recovery from HT than from Brucella agar (results not shown).

The eSwab is an all-in-one collection device that was shown to provide equal or superior release, viability, and recovery performance for 48 h at room temperature and 4°C with the most fastidious anaerobic bacteria compared to the conventional anaerobic transport system consisting of a rayon swab and an anaerobic transport tube. In addition, the eSwab provides the added ability to be used in automated specimen-plating devices.
